# Preoperative detailed evaluation intracranial artery stenosis using three-dimensional visualization analysis reduces the invasiveness of superficial temporal artery-middle cerebral artery bypass

**DOI:** 10.20407/fmj.2022-022

**Published:** 2022-12-27

**Authors:** Riki Tanaka, Fuminari Komatsu, Kento Sasaki, Kyosuke Miyatani, Yasuhiro Yamada, Yoko Kato, Yuichi Hirose

**Affiliations:** 1 Department of Neurosurgery, Fujita Health University Bantane Hospital, Nagoya, Aichi, Japan; 2 Department of Neurosurgery, Fujita Health University Hospital, Toyoake, Aichi, Japan

**Keywords:** Bypass surgery, Cerebral ischemia, Preoperative planning

## Abstract

**Objectives:**

Superficial temporal artery (STA) to middle cerebral artery (MCA) bypass surgery is a common treatment for preventing cerebral ischemia in patients with intracranial artery stenosis. The aim of this study was to analyze the surgical outcomes of the STA-MCA bypass procedure, particularly with regard to the invasiveness of targeted bypass (TB) with preoperative planning using Amira^®^ software.

**Methods:**

Consecutive patients with single STA-MCA bypass performed by a single neurosurgeon from January 2019 to May 2022 were included. The clinical parameters of seven TB patients were compared with those of 11 patients treated with the conventional method (CM).

**Results:**

Compared with CM patients, TB using Amira^®^ software patients had a shorter scalp incision (median [interquartile range]=11.2 [9.7–12.7] cm vs. 16.9 [16.0–17.7] cm, respectively; *p*=0.004], smaller craniotomy size (11.8 [11.5–14.4] cm^2^ vs. 20.9 [17.1–22.2] cm^2^, respectively; *p*=0.01], shorter surgery duration (201 [195–218] min vs. 277 [229–310] min, respectively; *p*=0.003], and less intraoperative bleeding (10 [10–20] g vs. 23 [20–50] g, respectively; *p*=0.033]. However, there were no differences in surgical complications between the two groups.

**Conclusions:**

Detailed preoperative evaluation using Amira^®^ software can reduce the invasiveness of the STA-MCA bypass procedure.

## Introduction

Superficial temporal artery (STA) to middle cerebral artery (MCA) bypass surgery is one of the most common cerebrovascular bypass procedures.^1^ Conventionally, neurosurgeons study preoperative three-dimensional (3D) digital subtraction angiogram (DSA) images for preoperative planning of the surgical procedure.^2^ In conventional bypass procedures, a larger scalp incision is made to allow the maximum length of the STA branch to be harvested. The most suitable recipient MCA branch is usually selected intraoperatively.^3^ Hence, a larger craniotomy may be required. Amira^®^ is a 3D visualization and analysis software(Thermo Scientific Co, Massachusetts, USA) that can be used to integrate 3D angiogram images of the six major intracranial vessels visualized by DSA.^4^ This software can also be used to integrate DSA images with other imaging data, such as from computed tomography (CT) angiography, CT 3D bone reconstruction, magnetic resonance imaging, and magnetic resonance angiography. The use of Amira^®^ software in preoperative planning allows neurosurgeons to identify the site where the STA branch (donor) and the MCA (recipient) are closest to each other. This allows a more targeted and smaller craniotomy to be performed for the bypass procedure. For a single surgical bypass procedure such as STA-MCA bypass, a targeted mini-craniotomy is preferred to reduce the surgical footprint. Although we previously described an initial technical report of targeted bypass (TB), the clinical significance of TB remains unclear.^5^ Thus, the aim of this study was to compare the surgical outcomes between the conventional method (CM) and TB.

## Methods

Consecutive patients with single STA-MCA bypass performed by a single neurosurgeon from January 2019 to May 2022 were included in this study. Eighteen bypasses in 18 patients (7 TB patients, 11 CM patients) were analyzed. The scalp incision length (cm), craniotomy size (cm^2^), surgery duration (minutes), intraoperative bleeding (g), and postoperative complications were analyzed. The scalp incision length (i.e., the line of staples on the surgical site) and craniotomy size were measured on postoperative 3D-CT images reconstructed with image analysis software (Ziostation2; ZIOSOFT, Tokyo, Japan). Measurements by Ziostation2 required manual operation.

All procedures performed in this study involving human participants were conducted in accordance with the latest version of the Declaration of Helsinki. Informed consent was obtained from all the participants included in the study. The institutional ethics committee approved this study.

### Statistical analysis

Because of the small sample size, data are presented as median and interquartile range (IQR). Categorical data are expressed as numbers. In univariate analysis, Pearson’s chi-square test or Fisher’s exact test with Yates’ correction were used to compare differences in categorical variables between the two groups, as appropriate. For continuous variables, comparisons between the two group were performed using the Mann–Whitney U test. Statistical analyses were performed using statistical software (SPSS v27; SPSS Japan Inc., Tokyo, Japan). A p-value of <0.05 (two-tailed) was considered statistically significant.

## Results

Eighteen single STA-MCA bypasses from 18 patients were included in this study. The median patient age was 62.5 years (IQR, 49.8–73.3 years). There were an equal number of men and women. The median body weight was 61.6 kg (IQR, 52.3–69.0 kg), and the median body mass index was 22.2 kg/m^2^ (IQR, 20.2–24.9 kg/m^2^). The other parameters of all patients are shown in Table 1.

**Figure 1:**
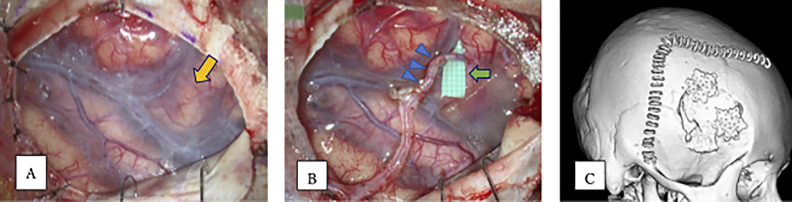
Intraoperative image of cerebrovascular bypass using the conventional method. (A) Image showing the frontal and temporal lobes bordered by the Sylvian fissure following exposure via a wide craniotomy using the conventional method. The orange arrow shows the recipient artery (middle cerebral artery [MCA]). (B) The blue arrowheads indicate the parietal branch (donor artery) of the superficial temporal artery (STA), which was to be anastomosed to the MCA (recipient artery) on the frontal lobe side. The flat rubber piece (green arrow) just below the MCA has a scale of 1 mm. (C) Image of the skull showing a large skin incision (staple line) and a large craniotomy using the conventional method.

Postoperative 3D CT scan images showed significantly shorter scalp incisions (i.e., the number of scalp staplers used) and a relatively smaller craniotomy in TB patients compared with CM patients (Figure 1). Furthermore, compared with CM patients, TB using Amira^®^ software patients had a significantly shorter scalp incision length (*p*=0.004), smaller craniotomy size (*p*=0.01), shorter surgery duration (*p*=0.003), and less intraoperative bleeding (*p*=0.033). However, there were no significant differences in postoperative complications, with complications seen in three subjects in the CM group (27%) and two subjects in the TB group (29%) (*p*=1.00; Table 2).

## Discussion

In single STA-MCA bypass using the CM, the scalp incision length typically depends on the length of the parietal branch of the STA. In this method, because the bypass site on the recipient artery (MCA branch) is usually not determined preoperatively, the scalp is cut along the STA branch until its end—this allows the maximal length to be harvested for the bypass.^6^ For the same reason, the typical craniotomy using the CM involves a larger pterional craniotomy to allow for exposure of the sylvian fissure and the MCA branch.^7^ Harvesting the maximal STA length ensures that this is adequate for the bypass procedure, irrespective of the graft site location on the recipient artery. Thus, CM does not align with the principle of minimally invasive neurosurgery, which involves minimizing the surgical footprint to create a small, but adequate, exposure for a safe and efficient procedure, and with minimal or no neural tissue retraction.^8,9^

**Figure 2:**
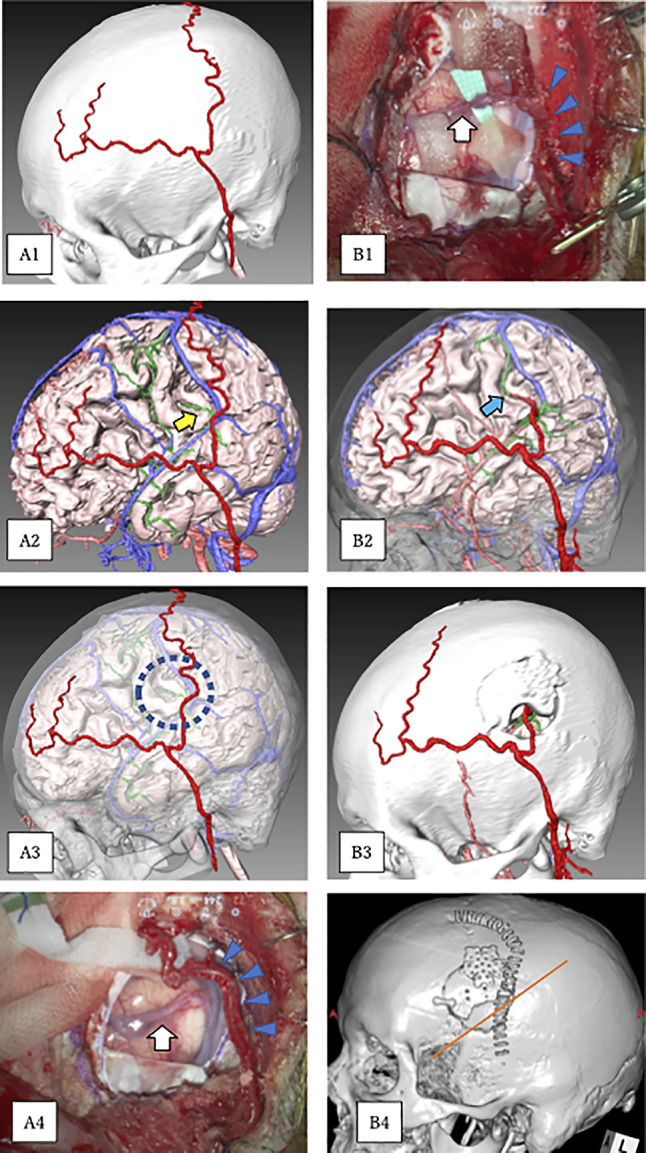
Preoperative and postoperative three-dimensional (3D) images constructed by Amira^®^ software. (A1) Preoperative 3D skull bone image showing the superficial temporal artery (STA). (A2) Image after digital removal of the skull bone structure from Figure A1. The cerebrum, intracranial arteries (pink), intracranial venous system (blue), and STA (red) are displayed. The middle cerebral artery (MCA) distal to the M1 stenosis is shown in green. The yellow arrow indicates the part of the M4 in the ischemic area that was closest to the parietal branch of the STA (donor artery), suggesting a possible candidate site on the recipient artery. (A3) A translucent skull bone image mounted on the image from Figure A2. The dotted circle shows the preoperatively planned site of the minimal craniotomy. (A4) Image of the actual intraoperative view just prior to bypass. Because of the small craniotomy, only the frontal lobe was exposed. The white arrow indicates the recipient M4. The blue arrowheads indicate the donor STA. (B1) An intraoperative image taken immediately after the bypass. The donor STA (blue arrowheads) was anastomosed to the recipient M4 artery (white arrow). The triangular green rubber piece below the M4 has a scale of 1 mm. (B2) Postoperative 3D image revealing successful STA-MCA anastomosis (blue arrow) at the targeted M4 site. (B3) Postoperative 3D skull bone image showing that the minimal craniotomy performed was same as the preoperatively planned craniotomy. (B4) The orange line representing the Sylvian fissure on a postoperative 3D skull bone image shows that the minimal craniotomy was placed on the frontal lobe without exposing the temporal lobe. Note that the length of the skin incision was shorter, the craniotomy size was smaller, and the brain was less exposed than for the conventional method (see Figure 1). These data are summarized in Table 2.

To ensure a minimal but optimal surgical site exposure for a single STA-MCA bypass, preoperative planning is essential for identifying the site where the donor (STA) and recipient (MCA branch) arteries are closest to each other. This is usually indicated by the crossing point between the STA branch and the MCA branch in a lateral view of vascular studies (Figure 2). In the present study, the Amira^®^ software was used preoperatively to identify this crossing point in all seven patients in the TB group. This software integrates DSA images with CT bone setting images, and displays the crossing point anatomically in relation to the bone surface. This enables the neurosurgeon to identify the best location for the bypass procedure, allowing a tailored craniotomy. To the best of our knowledge, there are no other reports using Amira^®^ software for STA-MCA bypass procedures.

In the present study, the Amira^®^ TB method required a smaller craniotomy for the single STA-MCA bypass procedure compared with the CM—the median tailored craniotomy size was 11.8 cm^2^ compared with 20.9 cm^2^ (diameter, 2.58 cm) for conventional craniotomy. For the harvested STA branch length, identification of the anastomosis site prior to surgery with TB allowed preselection and measurement of the desired STA branch length for the bypass. This allows a tailored scalp incision of the desired length to be made, eliminating excessive scalp incisions. Indeed, in the present study, a shorter scalp incision (median length, 11.2 cm) with TB was required for the single bypass procedure compared with CM (median length, 16.9 cm). A shorter scalp incision and a smaller craniotomy usually translates to a shorter surgery time and less intraoperative bleeding. Indeed, the median surgical time with TB was only 201 minutes compared with 277 minutes with the CM. Additionally, there was significant reduction in intraoperative bleeding with TB (median intraoperative bleeding, 10 g) compared with the CM (median intraoperative bleeding, 23 g). By contrast, there were no differences in the complication rates for the single STA-MCA bypass procedures between the two groups, indicating that the tailored surgical procedure was not inferior to the CM.

A similar tailored approach using 3D angiography was described by Nakagawa et al.^10^ However, a disadvantage of 3D angiography alone is the inability to modify the images to improve visualization. In our study, the Amira^®^ software enabled us to modify the images by selecting a specific vessel of interest and labelling it accordingly. Fischer et al. also reported the use of 3D virtual reality software (Dextroscope) to fuse 3D magnetic resonance angiography and CT images.^11^ In our study, 3D DSA images were integrated by image fusion for preoperative planning. Comparisons of the advantages, disadvantages, skin incision length, and craniotomy diameter between the Amira^®^ method and other reported preoperative simulations are shown in Table 3. Similarities between the methods included the use of comprehensive images of the appropriate donor and recipient vessels, the ability to determine ideal skin incisions, and the use of small craniotomies. However, our TB method was associated with a larger average craniotomy size and skin incision length. This is because we performed a longer STA dissection than the other studies. Because the STA may contain local calcifications and thin branches that are unsuitable for bypass, we avoided these regions and dissected the STA as long as possible for a reliable bypass procedure.

Numerous studies have reported the advantages of minimally invasive cranial surgery.^12–16^ In a review esthetic outcomes of conventional pterional craniotomy with ‘mini-pterional’ and ‘nano-pterional’ procedures, alternative minimally invasive techniques were associated with reduced temporalis muscle atrophy, facial nerve weakness, and scar formation compared with conventional pterional craniotomy.^17^ Minimally invasive surgery is very difficult to define, and there is no consensus on a quantitative measure. Therefore, we did not collect data on the preoperative and postoperative physical burdens of the procedures on the patients and their inflammatory responses. Collection of these data are required in future studies to more accurately define ‘minimally invasive’. We also believe that data on the duration of postoperative symptoms and drug use for their treatment are necessary for short-term evaluation. Furthermore, longer term outcomes examining the efficacy of the two bypass surgery methods for preventing cerebral infarction are required.

## Conclusion

Compared with CMs, using Amira^®^ software helps in TB by significantly reducing the duration of surgery and allowing a shorter scalp incision and smaller craniotomy, resulting in less intraoperative bleeding. Thus, we strongly recommend the use of Amira^®^ software for preoperative assessment of the optimal site for surgical bypass to avoid an unnecessarily large scalp incision or a large craniotomy for single STA-MCA bypass procedures.

## Figures and Tables

**Table1:** Patients’ backgrounds and surgical outcomes

Parameter	Median/Proportion (n=18)*
Age (year)	62.5 (49.8–73.3)
Sex (male/female)	9/9
Height (cm)	164.5 (155.3–170.8)
Body weight (kg)	61.5 (52.3–69.0)
BMI (kg/cm)	22.1 (20.2–24.9)
Surgery time (min)	228 (205–301)
Intraoperative bleeding (g)	20 (10–43)
Skin incision length (cm)	15.9 (11.1–17.5)
Craniotomy area (cm^2^)	18.1 (11.8–21.3)
Postoperative complications (%)	5 (28%)

* Data are presented as median (interquartile range [25th–75th percentile]). BMI, body mass index.

**Table2:** Comparison of patients’ backgrounds and surgical outcomes between the conventional method and the target bypass with Amira^®^ software groups

	Conventional method (n=11)**	Target bypass method (n=7)**	P-value
Age (year)	52 (47–70)	72 (54–78)	0.037
Sex (male/female)	4/7	5/2	0.355
Height (cm)	165 (153–175)	164 (160–167)	0.821
Body weight (kg)	62 (50–68)	61 (53–72)	0.650
BMI (kg/cm)	22.1 (19.2–24.9)	22.1 (20.8–25.8)	0.298
Surgery time (min)	277 (229–310)	201 (195–218)	0.003
Intraoperative bleeding (g)	23 (20–50)	10 (10–20)	0.033
Skin incision length (cm)	16.9 (16.0–17.7)	11.2 (9.7–12.7)	0.004
Craniotomy area (cm^2^)	20.9 (17.1–22.2)	11.8 (11.5–14.4)	0.010
Postoperative complications (%)	3 (27%)	2 (29%)	1.000

** Data are presented as median (interquartile range [25th–75th percentile]). BMI, body mass index.

**Table3:** Advantages, disadvantages, skin incision length, and craniotomy diameter in several studies with preoperative simulations compared with the Amira^®^ method for a single STA-MCA bypass

	Method	Advantages	Disadvantages	Skin incision	Craniotomy sides
Nakagawa et al., 2010	3D rotational reconstruction DSA (n=28 subjects, single arm study)	Excellent images of small arteries with a diameter of <1 mm	No planning software; cannot fuse 3T MR angiographic and high-resolution 3D CT images	Mean=5.4 cm (range=4–6.5 cm)	*Craniotomy diameter*: mean=22 mm (range=17.5–27.5 mm)

Fischer et al., 2009	3T MR angiography data (n=5 subjects, single arm study)	3D virtual reality planning tool (Dextroscope); can merge MR with CT images	Cannot clearly identify small appropriate recipients; cannot produce information about the skull	Mean=4.6 cm (range=4.4–5.0 cm)	*Craniotomy diameter*: mean=22 mm (range=20–25 mm)

Tanaka et al., 2022 (current study)	3D rotational reconstruction DSA and CT scan (n=7 subjects with control arm of n=11 subjects)	3D virtual reality planning tool (Amira^®^); can merge 3D DSA with CT and MR images; can modify the images to mark area of interest.	Unable to simulate the blood flow in the donor and recipient following bypass procedure	Median=11.2 cm (range=9.7–12.7 cm)	*Craniotomy area*: mean=11.8 cm^2^
